# Integrative Multivariate Analysis of Milk Biomarkers, Productive Performance, and Animal Welfare Indicators in Dairy Cows

**DOI:** 10.3390/ani15213202

**Published:** 2025-11-03

**Authors:** Daniela Elena Babiciu, Florin Ioan Beteg, Mihai Cenariu, Anamaria Blaga Petrean, Sorin Marian Mârza, Eva Andrea Lazar, Silvana Popescu

**Affiliations:** 1 Faculty of Veterinary Medicine, University of Agricultural Sciences and Veterinary Medicine Cluj-Napoca, 400372 Cluj-Napoca, Romania; 2 Horse Welfare Association, 407207 Feiurdeni, Romania

**Keywords:** welfare assessment, dairy cows, milk composition, welfare indicators

## Abstract

**Simple Summary:**

Consumers, veterinarians, and farmers are increasingly concerned about animal welfare on dairy farms. Thus, invasive procedures should be avoided along the production flux and also during additional procedures, such as performing welfare measurements. In this study, we investigated whether routinely collected milk composition data could serve as an indicator of cows’ health and well-being. Data from 37 commercial farms were analysed, combining milk composition with direct assessments of health, behaviour, and housing conditions. Our findings indicated that specific milk components, such as the fat-to-protein ratio, energy-related metabolites, and udder health indicators, were significantly associated with markers of favourable or unfavourable welfare. Farms with healthier and more content cows tended to produce milk with a better fat-to-protein balance and lower evidence of disease. Conversely, farms with welfare challenges such as lameness, mastitis, or poor hygiene exhibited distinctive milk profiles. These results demonstrated that routine milk testing, already an integral part of herd management, can provide a simple and non-invasive means of monitoring animal welfare. Using milk characteristics to explore the cows’ health and welfare, farmers and advisors can make quicker, evidence-based decisions that benefit both the animals and society.

**Abstract:**

Animal welfare is increasingly recognised as a core component of sustainable dairy production, yet objective assessment at the herd level remains challenging. This study evaluated whether milk biomarkers can serve as non-invasive indicators of cow welfare. Thirty-seven dairy farms were assessed using the Welfare Quality^®^ protocol and various milk analysis parameters. As a first line of results, Spearman correlations revealed strong associations between milk biomarkers and welfare indicators. For example, a higher fat-to-protein ratio was linked to better feeding, lower prevalence of hunger, and improved human–animal relationships. In contrast, elevated somatic cell count and differential somatic cell count were associated with mastitis, lameness, dirtiness, and reduced emotional well-being. Using Principal Component Analysis (PCA), three dimensions were identified, health–hygiene, socio-behavioural, and metabolic stress, explaining 44.7% of variance. K-means clustering distinguished three herd profiles: feeding–metabolic balance, behavioural–comfort, and clinical–hygiene risk. These findings demonstrated that routine milk biomarkers provide integrated, non-invasive information on herd health, behaviour and, comfort. Incorporating routine milk analysis into welfare assessments can support the early detection of issues, facilitate evidence-based decision-making, and promote sustainable dairy management.

## 1. Introduction

Milk is an ideal matrix for cattle welfare monitoring because it is easily sampled and reflects the interplay of diet, metabolism, and udder health. Its biochemical, cellular, and spectral biomarkers provide insight into the physiological condition, metabolic balance, and immune response, key components of overall welfare [1]. Produced by mammary epithelial cells, milk contains fat, protein, lactose, minerals, enzymes, cells, and metabolites influenced by genetics, diet, physiology, and disease [2,3]. Strong blood–milk correlations support the use of milk traits as indicators of systemic status, particularly during the transition period [4].

Among metabolic indicators, the fat-to-protein ratio (FPR) is a well-established marker of energy balance. Elevated values indicate lipomobilisation and risk of ketosis, while low ratios suggest subacute ruminal acidosis [5,6]. FPR reliably reflects metabolic disorders [7] and correlates negatively with energy balance, especially in the postpartum period [5]. It also responds to environmental stressors, such as heat [8,9], and differs between farms with contrasting welfare standards [10].

β-hydroxybutyrate (BHB), the main ketone body, directly indicates lipolysis and hepatic ketogenesis. Elevated milk or blood BHB is associated with reduced feed intake, impaired immunity, and postpartum disorders [11,12]. Despite variations with lactation stage, MIR-predicted BHB enables accurate herd-level monitoring [13,14,15], and metabolomic studies confirm its role as a marker of metabolic disease and mastitis risk [16].

Milk urea nitrogen (MUN) reflects protein–energy synchrony and nitrogen utilisation efficiency, varying with diet and energy intake [17,18]. While mainly a nutritional indicator, it also relates to reproduction and nitrogen losses [19,20,21], though interpretation depends on the environment and lactation stage [17,18].

Udder health, a key welfare component, is routinely assessed using somatic cell count (SCC) and differential SCC (DSCC), which provide detailed immune profiling [22]. Combined SCC-DSCC monitoring enhances mastitis detection and accounts for parity and lactation stage [23,24,25,26]. DSCC correlates strongly with milk quality and udder health, confirming its value in herd monitoring [27,28].

Lactose concentration indicates udder integrity, decreasing during mastitis or epithelial damage [29,30]. It is associated with lower SCC and better metabolic status [8,31,32], while pathogen-specific mastitis and inflammation reduce lactose content and yield [33,34]. Behavioural studies further link lactose levels to emotional states, highlighting its potential as a physiological and welfare marker [35].

Microbiological traits, including total bacterial count (TBC), mainly reflect hygiene. Although their correlations with welfare indices are weak [36,37], herd-level variation is related to management and housing [38]. Routinely collected herd data (RHD), such as production or fertility, have been proposed as welfare proxies [39,40,41] but cannot replace animal-based measures; milk yield alone inconsistently reflects welfare [42].

Despite extensive research on single biomarkers, their combined ability to reflect herd-level welfare, including feeding, housing, health, and behaviour, remains underexplored. Advances in mid-infrared spectroscopy, in-line sensors, and data science now allow for the integration of metabolic, inflammatory, and hygiene signals with animal-based measures [14,43]. Therefore, this study aimed to (1) investigate the relationships between milk biomarkers and animal-based welfare indicators; (2) identify multivariate patterns integrating health, metabolism, and behaviour; and (3) evaluate whether these patterns can classify farms into distinct welfare categories.

Unlike previous studies that investigated individual production or health traits, the present work integrates multiple milk biomarkers with animal-based welfare indicators using a multivariate approach at the herd level, offering a novel framework for precision welfare assessment.

## 2. Materials and Methods

### 2.1. Study Population

The study was conducted between April 2023 and December 2024 on 37 commercial dairy farms in Transylvania, Romania, comprising a total of 3377 lactating cows. Herd size ranged from 13 to 340 animals, predominantly Holstein Friesian (78%) and Romanian Spotted (22%) breeds, and encompassed a diversity of housing and management systems, including free-stall (26) and tie-stall (11) configurations, with variable access to pasture and/or outdoor loafing areas (OLAs). The farms were recruited with the assistance of breeders’ associations and field veterinarians. Initially, 54 farms were identified; however, some owners later declined participation (11 farms). Inclusion criteria were commercial dairy herds with ≥10 lactating cows; willingness of the farm owner to participate; and accessibility for repeated welfare and milk sampling. Farms with incomplete welfare or milk records were excluded from the analysis (6 farms). As the study period spanned multiple seasons, potential seasonal variations (e.g., access to pasture, heat stress) were inherently included in the dataset. A particular strength of this study was the collection methodology of all cow-based parameters, which were sampled individually.

### 2.2. Welfare Assessment

Animal welfare was assessed twice, at one-month intervals, using the Welfare Quality^®^ protocol for dairy cattle [44]. For each farm, the results from the two evaluations were averaged to obtain a final composite welfare score. The protocol integrates both resource- and animal-based indicators to provide a comprehensive assessment of feeding, housing, health and behaviour, covering four principles (good feeding, good housing, good health, and appropriate behaviour) subdivided into 12 criteria (absence of prolonged hunger and thirst, comfort around resting, ease of movement, absence of injuries, absence of disease, absence of pain induced by management procedures, social and other behavioural expressions, human–animal relationship, and positive emotional state) and further into multiple measures.

The individual data collection for 3377 lactating cows included measures such as the identification of very lean cows, lying behaviour evaluation (duration of lying down movements, lying down with collisions, lying position), cleanliness scores (dirty lower legs, dirty udder, dirty flanks and upper legs), avoidance distance tests, recording of social interactions (butts and displacements), and the clinical signs of health issues (nasal discharge, increased respiratory rate, ocular discharge, diarrhoea, vulvar discharge, coughing, and integument alterations, such as hairless patches, lesions/swellings). Lameness was assessed individually as well (scoring each cow as non-lame, moderately lame, or severely lame), and using qualitative behaviour assessment (QBA), each cow was scored on the protocol’s pre-established terms (active, relaxed, fearful, agitated, calm, content, indifferent, frustrated, friendly, bored, playful, positively occupied, lively, inquisitive, irritable, uneasy, sociable, apathetic, happy, and distressed) [44]. By consulting farm records, the prevalence of mastitis, dystocia, downer cows, and mortality was registered for each facility. The detailed assessment methodology is described in the Welfare Quality^®^ Assessment protocol for cattle [38]. For the calculation of the criteria and principle scores, the Welfare Quality^®^ scoring system software was used [45].

All the procedures involving animals were carried out in accordance with the ethical guidelines of the Romanian National Animal Protection Law [46].

### 2.3. Milk Sampling and Laboratory Analysis

To ensure direct comparability between welfare status and milk quality, individual milk samples were collected from all lactating cows on each farm at the time of welfare assessment. Sampling was performed during the morning milking. The collection of samples (a total of 6754) and their analysis were carried out twice, at one-month intervals, in parallel with the two welfare assessments. For each farm, the results obtained from all cows were averaged to yield a single monthly value for each milk quality parameter. The two monthly means were then averaged to generate a final farm-level result.

The variables included compositional traits, such as fat, protein, casein, and lactose contents, from which the fat-to-protein ratio (FPR) was calculated. The milk yield was expressed as the average quantity of milk obtained per cow per milking. In addition, metabolic biomarkers including urea, acetone, and β-hydroxybutyrate (BHB) were measured to capture aspects of protein–energy balance and ketone body production. Udder health was assessed using somatic cell count (SCC) and differential somatic cell count (DSCC), whereas hygienic quality was evaluated using total plate count (TPC).

All physical–chemical parameters, as well as SCC and DSCC, were determined using a CombiFoss™ 7 FT analyser (Foss Electric, Hillerød, Denmark), whereas TPC was quantified with a BactoScan™ FC analyser (Foss Electric, Hillerød, Denmark). All laboratory determinations were performed in an ISO/IEC 17025-accredited laboratory (RENAR, Bucharest, Romania), following the International Organisation for Standardisation (ISO) procedures.

### 2.4. Statistical Analysis

All statistical analyses were conducted in R software (version 4.5.1, R Core Team, Vienna, Austria) using the packages tidyverse, psych, cluster, factoextra, pheatmap, mclust, writexl, and ggplot2. Descriptive statistics were computed for all variables to summarise central tendencies and variation across farms. Pairwise associations between milk biomarkers, production parameters, and welfare indicators were assessed using Spearman’s rank correlation, a robust method for non-normal distributions. To reduce the risk of type I errors, *p*-values were adjusted for multiple testing using the false discovery rate (FDR) method. Post hoc power estimation indicated that the achieved power exceeded 0.90 for moderate correlations (rs ≥ 0.40; α = 0.05, two-tailed, *n* = 37 herds).

Before Principal Component Analysis (PCA) and K-means clustering, all variables were standardised to z-scores to ensure comparability across different measurement scales. PCA was used to reduce dimensionality and identify latent multivariate patterns. The Kaiser–Meyer–Olkin (KMO) measure indicated sampling adequacy (>0.6), and Bartlett’s test was significant (*p* < 0.001), confirming sufficient inter-variable correlations to justify the use of PCA. PCA loadings, explained variance, and component scores were extracted, then biplots were generated to visualise relationships between variables and farms.

Cluster analysis was performed using two complementary approaches. First, K-means clustering was applied to the standardised dataset to classify farms with similar milk composition and welfare profiles. The optimal number of clusters (k) was determined using the Silhouette coefficient, Calinski–Harabasz index, and Gap statistic. Second, hierarchical agglomerative clustering (Ward’s linkage, Euclidean distance) grouped variables to identify domains of co-varying biomarkers and welfare indicators, visualised as a clustered heatmap.

## 3. Results

### 3.1. Descriptive Data

#### 3.1.1. Milk Biomarkers and Production

A comprehensive overview of milk composition, metabolic status, udder health, and production performance across the 37 studied farms is presented in Table 1.

#### 3.1.2. Welfare Indicators

The results of the welfare assessment are shown in Table 2, Table 3 and Table 4. The assessment of the 37 farms using the Welfare Quality^®^ protocol revealed considerable variation across principles, criteria, and measures.

“Good feeding” and “Good housing” principles (Table 2) showed moderate scores, with issues related to body condition and housing hygiene (Table 3). The “Good health” principle achieved the lowest values due to the high prevalence of mastitis, diarrhoea, and integument alterations (Table 3). Appropriate behaviour presented heterogeneous outcomes, with well-expressed social behaviours but limited exploratory and play behaviours.

The Qualitative Behaviour Assessment (Table 4) indicated predominantly positive emotional states, although variability was observed between farms.

### 3.2. Associations Between Milk Biomarkers and Animal-Based Welfare Parameters

Table 5 presents the significant correlations found between milk biomarkers and welfare outcomes.

Higher FPR was linked to improved feeding, lower disease prevalence, and better human–animal relationships, while elevated BHB was associated with poorer comfort and more injuries. Milk yield correlated positively with housing and health and negatively with mortality and dirtiness. SCC and DSCC were consistently linked to mastitis, lameness, and negative emotional states, whereas higher lactose concentrations were associated with good health and inquisitive, calm behaviour. As shown in Table 5, the correlations found were, in most cases, moderate (r_s_ between 0.44 and 0.57).

### 3.3. Multivariate Structure

Principal Component Analysis (PCA) was applied to explore the multivariate structure of welfare indicators, milk biomarkers, and production traits across the 37 farms. The scree plot and eigenvalue distribution supported retention of the first three components, which together explained 44.7% of the total variance (PC1 = 24.2%, PC2 = 11.8%, PC3 = 8.7%) (see Figure S1 in the Supplementary Material). Although the cumulative variance did not reach the conventional 60–70% threshold typically considered sufficient for robust factor interpretation, the retained dimensions nevertheless revealed biologically meaningful patterns.

Because loadings ≥ |0.30| are usually considered relevant for interpretation [47], we initially used this threshold but adopted a more inclusive criterion (≥|0.10|) given the exploratory nature of the study and the relatively low variance explained per component. This approach allowed identification of subtle but biologically relevant associations while acknowledging the hypothesis-generating character of the analysis. PC1 (24.2% variance explained) represented a broad welfare-health gradient. Negative loadings were associated with favourable conditions, including “Good health”, “Good housing”, “Good feeding”, and positive emotional states (calm, content, happy). Positive loadings reflected adverse welfare outcomes such as elevated SCC and DSCC, mastitis prevalence, lameness, integumentary alterations, cow dirtiness, diarrhoea, nasal discharge, mortality, and increased lying down collisions. This component captured the contrast between herds with adequate metabolic balance, udder health, and emotional well-being versus those with disease burden, hygiene problems, and reduced comfort. These findings were consistent with the Spearman correlations, where SCC and DSCC were strongly associated with disease, lameness, and hygiene deficits. PC2 (11.8% variance explained) described a socio-behavioural and relational axis. Positive loadings included FPR, fat percentage, good human–animal relationship, “Good feeding”, “Appropriate behaviour”, and positive QBA states (lively, active, content, relaxed, playful). Negative loadings reflected poor housing, comfort deficits, injuries, diarrhoea, and negative emotional states (fearful, agitated, irritable, and distressed). The variable “cows that can be touched” loaded strongly on the positive side, linking metabolic balance (FPR) with better human–animal interaction quality. This axis differentiated herds with good stockmanship, balanced feeding, and positive affective states from those with housing deficits and signs of behavioural distress.

**Figure 1:**
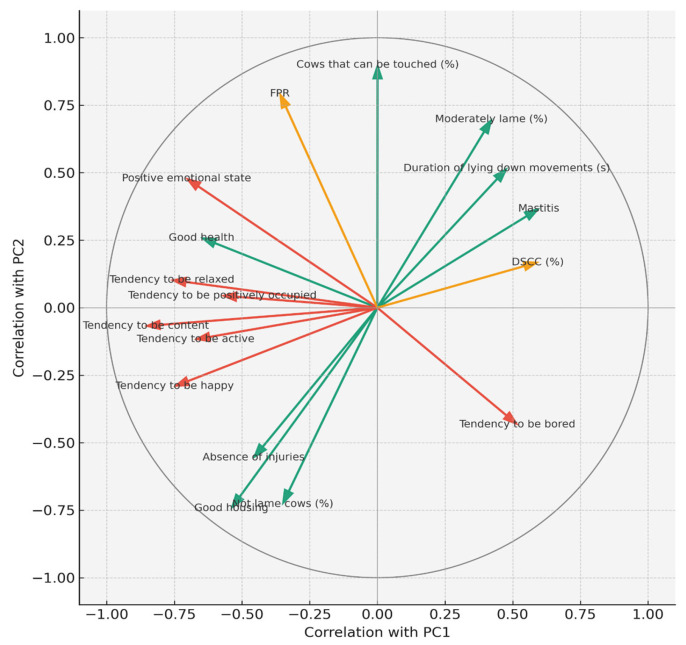
PCA correlation circle (PC1 vs. PC2) showing the contribution of milk biomarkers, production traits, and welfare indicators to the first two components. Vector direction indicates association, and length indicates contribution strength. The colours of the vectors are green = health and housing-related welfare indicators, red = emotional/affective indicators, and orange = milk biomarkers.

Figure 1 shows the PCA correlation circle (PC1 vs. PC2), which graphically represents these relationships by illustrating how milk biomarkers, production traits, and welfare indicators contribute to the first two principal components. Vectors pointing in the same direction indicate positive associations, those in opposite directions indicate negative associations, and vector length reflects the strength of the contribution to PC1 and PC2.

PC3 (8.7% variance explained) captured a bipolar dimension reflecting a trade-off between metabolic stress and positive welfare factors. Positive loadings were driven by BHB, acetone, milk fat, integument alterations, dirty legs, and ambivalent behavioural traits (playful, fearful, and agitated). Negative loadings reflected good human–animal relationships, expression of social behaviours, absence of disease and injuries, and positive affective states (calm, friendly). This dimension distinguished herds experiencing metabolic stress and hygiene problems from those with better health, improved stockmanship, and calmer social dynamics. These results reinforced the Spearman findings linking BHB with injuries, resting discomfort, and dystocia while highlighting the complementary value of behavioural and relational indicators. In summary, the PCA highlighted three biologically relevant dimensions: health and hygiene (PC1), socio-behavioural dynamics (PC2), and metabolic stress (PC3) that together explained nearly half of the variance in the dataset. These axes confirmed that welfare status in dairy herds results from the interplay between metabolic balance, udder health, housing, and behavioural expression.

K-means clustering was performed on the standardised dataset to identify groups of farms with similar milk composition, production traits, and welfare profiles. The optimal number of clusters (k) was evaluated using three complementary methods: the Calinski–Harabasz index (Figure S2A, see the Supplementary Material), average silhouette width (Figure S2B in the Supplementary Material), and the gap statistic (Figure S2C in the Supplementary Material). Both the Calinski–Harabasz and silhouette indices indicated that k = 2 provided the most compact and well-separated solution, whereas the gap statistic suggested a more granular structure with k = 6. To ensure biological relevance and practical interpretability, a three-cluster solution (k = 3) was selected as a compromise, balancing statistical separation with the need to capture intermediate management–welfare profiles.

K-means clustering confirmed the PCA tendencies by partitioning farms into three coherent groups. Cluster 1 was the feeding–metabolic profile, characterised by high FPR, “Good feeding”, and “Appropriate behaviour”, suggesting that favourable protein–energy balance was associated with improved welfare outcomes. Cluster 2 was the behavioural–comfort profile, dominated by QBA descriptors (happy, lively, content, relaxed, sociable), “Good housing”, “Good health”, lactose, and milk yield, reflecting optimal cow comfort and behavioural expression. Cluster 3 was the clinical–hygiene risk profile, defined by SCC, DSCC, mastitis, diarrhoea, nasal discharge, dirtiness scores, and mortality, representing herds with compromised udder health and hygiene challenges.

**Figure 2:**
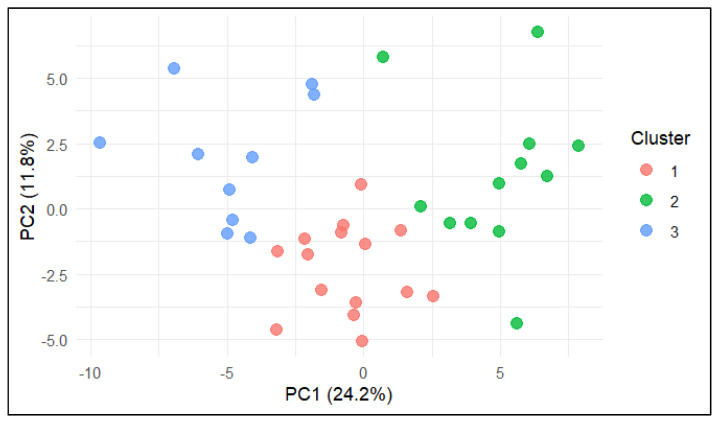
PCA biplot (PC1 vs. PC2) with farms coloured according to K-means cluster assignment. Cluster 1 = feeding–metabolic profile, Cluster 2 = behavioural–comfort profile, Cluster 3 = clinical–hygiene risk profile.

The distribution of farms in the PCA space, coloured according to K-means cluster assignment, is shown in Figure 2. Clear spatial separation between clusters was observed. Farms in Cluster 1 (red) were located around the origin and aligned with average feeding and production parameters; farms in Cluster 2 (green) were positioned toward the positive PC1 axis, associated with better welfare and behavioural scores; and farms in Cluster 3 (blue) occupied the negative PC1 axis, overlapping with indicators of udder health challenges and hygiene issues.

**Figure 3:**
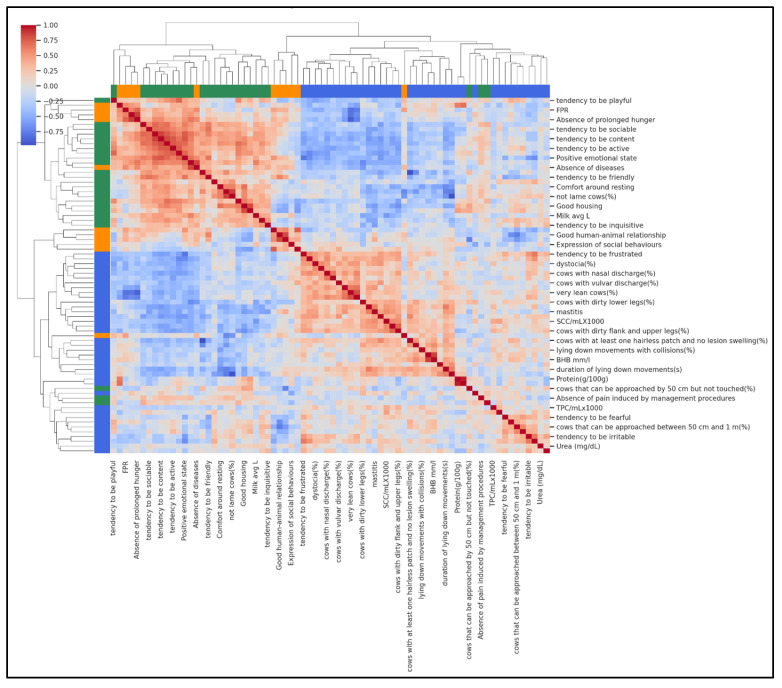
Hierarchical clustering heatmap showing how variables group together based on their similarity across farms. Each row represents one variable, and the branching tree (dendrogram) shows which variables are most closely related. Colours indicate the three main domains: green for metabolic–nutritional (feeding balance and milk traits), orange for behavioural–comfort variables (housing, comfort, positive behaviours), and blue for clinical–hygiene variables (udder health, dirtiness, mortality). Variables on the same branch share similar patterns, meaning that farms with high values for one variable often have high (or low) values for the others in the same group.

To complement the farm-level typologies identified by PCA and K-means, a hierarchical agglomerative clustering of variables was performed using Ward’s linkage and Euclidean distance. This analysis grouped milk biomarkers, production traits, and welfare indicators into three biologically meaningful domains, revealing how these variables co-varied across the 37 farms (Figure 3). The first domain represented a metabolic–nutritional profile, including FPR, milk fat, protein, and milk yield, capturing feeding balance and production level. The second domain reflected a behavioural–comfort profile, characterised by QBA descriptors, “Good housing”, “Good health”, and absence of injuries, indicating improved cow comfort and positive emotional states. The third domain represented a clinical–hygiene risk profile, grouping SCC, DSCC, mastitis, diarrhoea, dirtiness scores, and mortality, signalling farms with compromised udder health and hygiene issues.

Figure 3 shows the clustered heatmap with colour gradients representing pairwise correlations between variables (red = positive, blue = negative). The clear clustering pattern visually confirms that metabolic, behavioural, and health–hygiene variables tend to vary together, providing an integrated view of herd welfare profiles.

## 4. Discussion

This study explored whether routinely collected milk biomarkers can act as non-invasive indicators of dairy cow welfare. By integrating compositional, metabolic, and udder health traits with animal-based measures (ABMs) from the Welfare Quality^®^ protocol, consistent associations and multivariate patterns were identified, which provide new insights into how milk reflects the interplay of nutrition, housing, health, and behaviour. These findings strengthen the growing interest in using milk composition as a real-time diagnostic tool for welfare assessment in dairy systems [43,48].

### 4.1. Descriptive Patterns of Welfare and Milk Quality

The descriptive analysis of welfare indicators revealed considerable heterogeneity among the 37 dairy farms between principles and criteria, confirming that the dairy cows’ welfare level was strongly influenced by variations in management, infrastructure, and husbandry practices. “Good feeding” scores indicated that water availability was generally sufficient, but body condition was suboptimal on many farms, with more than 60% of cows classified as very lean in some cases. This suggests variability in the adequacy of nutritional management and feeding strategies, consistent with earlier studies showing that underfeeding remains a persistent welfare concern in small and medium-sized Eastern European dairy systems [49,50]. “Good housing” varied markedly across farms. Poor comfort around resting, evidenced by the high prevalence of dirty body regions, reflected deficiencies in bedding usage and stall design. This was in agreement with evidence linking hygiene-related body soiling to higher mastitis incidence and compromised udder health [38,51]. Conversely, high “Ease of movement” scores in many farms confirmed that adequate space and design can positively influence welfare.

The lowest results were obtained for the “Good health” principle, with considerable variation in lameness and mastitis prevalence. While severe lameness was relatively infrequent, moderate lameness affected more than 10% of cows, in agreement with previous European studies reporting locomotor disorders as a persistent welfare and economic issue [52,53]. The results on behaviour and emotional state highlighted notable differences in human–animal relationships and social behaviour expression. High scores for positive social interactions and QBA descriptors such as “calm,” “playful,” and “positively occupied” in some farms demonstrated the potential for welfare improvements through adequate management. However, the presence of negative descriptors in other farms indicated deficiencies in environmental conditions or human–animal interactions. These findings support previous research showing the direct impact of management factors on affective state and cattle behaviour [54].

Regarding milk composition, the main parameters (fat, protein, casein, and lactose) were within the physiological ranges, indicating relatively balanced nutrition and metabolic stability at the population level. However, the variability in milk urea levels suggested significant differences in protein balance and nitrogen utilisation across farms. Similar findings have been reported in recent studies linking excess MUN with reduced reproductive performance and environmental nitrogen losses [10,18]. Indicators of udder health revealed substantial variations between the studied farms. Elevated SCC and DSCC values in some herds pointed to subclinical mastitis and inflammatory challenges. These findings confirmed the value of combining SCC and DSCC in routine monitoring, as recent studies highlight DSCC as a sensitive predictor of udder inflammation and milk quality losses [27,28]. The large dispersion of TPC underscored the role of hygiene and equipment sanitation, with evidence showing that poor hygienic practices increase variability in both SCC and bacterial counts [38]. Milk yield per cow showed wide variation, likely reflecting genetic potential, nutrition, and housing conditions. Importantly, bulk tank studies demonstrate that higher-yielding herds often display better welfare indicators and improved udder hygiene [10], supporting the observed associations between productivity and welfare outcomes.

### 4.2. Associations Between Biomarkers and Welfare Indicators

This study revealed biologically meaningful associations between routinely collected milk biomarkers and animal-based welfare parameters, confirming their potential as non-invasive welfare indicators.

Fat-to-protein ratio (FPR) and β-hydroxybutyrate (BHB): indicators of metabolic balance

Spearman correlations revealed that FPR was strongly associated with favourable welfare outcomes, including improved feeding, reduced prevalence of very lean cows and diarrhoea, and better human–animal interactions. These findings support earlier evidence that FPR reflects nutritional balance and energy status [55] and also align with recent studies linking balanced FPR with improved welfare and bulk milk quality [10]. The strong negative associations between FPR and lean body condition, diarrhoea, and mortality highlighted its value for capturing both metabolic adequacy and welfare resilience [5,7]. Within its physiological range, FPR indicates adequate energy supply relative to protein intake, thereby reducing the risk of negative energy balance and metabolic stress [6,56]. Our findings were in line with studies showing that optimal FPR is associated with enhanced rumination, locomotion, and behavioural signs of comfort [7,57], suggesting that FPR reflects not only metabolic status but also the capacity of cows to express positive affective states.

Higher BHB levels were associated with lower comfort around resting scores and increased integumentary lesions, dystocia, and injuries. These relationships reflect the physiological strain of negative energy balance [13,14]. Similarly, previous studies link elevated BHB to ketosis, displaced abomasum, and reduced body condition [58]. Notably, our results indicated that elevated BHB not only signals metabolic imbalance but also manifests in welfare outcomes observable at the herd level, such as reduced comfort and impaired behavioural expression. Together, FPR and BHB form a robust two-component system for measuring metabolic resilience and identifying herds at risk of multiple welfare challenges early in lactation [15,43].

Indicators of udder health (SCC, DSCC, and lactose): connecting udder inflammation to welfare status

Both SCC and DSCC demonstrated wide-ranging associations with welfare outcomes. High SCC values were linked to mastitis, lameness, and dirtiness, while high DSCC was negatively related to positive QBA descriptors such as calmness, happiness, and sociability. These findings confirm the recognised role of SCC as a marker of intramammary infection [22,23] and highlight DSCC as a more sensitive tool for detecting subclinical udder infections [25,26]. Our results confirm that herds with elevated SCC/DSCC experienced wider welfare impairments, supporting evidence that mastitis exerts cumulative and prolonged negative effects on milk yield and lactose content [30].

The determined lactose values exhibited an inverse correlation with SCC and mastitis, consistent with the literature describing its decline during epithelial injury [30]. The positive association of lactose with “Good health”, absence of disease, and favourable emotional states (active, relaxed, lively) reinforced its value as a cost-effective, non-invasive biomarker for herd health and cow welfare [31,32]. More recently, lactose concentration has been directly correlated with calmer behavioural states and improved welfare, showing its role as both a physiological and behavioural indicator [35].

Together, SCC, DSCC, and lactose provide a comprehensive view of udder health by combining information on inflammatory burden, immune status, and secretory capacity, thereby improving the accuracy of welfare monitoring.

Milk yield and total plate count (TPC): indicators of productivity and hygiene

Higher milk yield was positively associated with good housing, good health, comfort while resting, and ease of movement, whereas lower milk yield correlated with udder dirtiness, skin alterations, and moderate lameness. These results suggest that especially the high-yielding herds benefit from superior comfort and housing conditions [42,59]. Milk yield also showed positive correlations with behavioural indicators, such as calmness, contentment, and liveliness, indicating that productivity and welfare can be complementary outcomes of good management [39].

Although TPC did not show statistically significant correlations after FDR correction, its numerical associations with poor hygiene indicators (udder dirtiness, lying down collisions, ocular discharge) suggest that it remains a valuable metric for assessing milking hygiene [25]. Bulk tank TPC values exceeding 100,000 CFU/mL are widely recognised as evidence of deficiencies in teat preparation, bedding hygiene, or equipment maintenance [36]. While not a direct welfare indicator, TPC remains an important management parameter with indirect welfare implications.

Milk urea nitrogen (MUN): a nutritional management metric

MUN did not show strong associations with welfare indicators but displayed a tendency to covariate with irritability and emotional reactivity, maintaining its potential to indicate protein–energy imbalance. High MUN commonly reflects excess rumen-degradable protein relative to fermentable energy [17,18], whereas very low MUN signals protein deficiency, which may compromise microbial protein synthesis [19]. These findings confirm that MUN is best interpreted as a nutritional management tool rather than a direct welfare indicator. Nonetheless, optimising MUN remains important for improving fertility, feed efficiency, and reducing nitrogen emissions [23,60].

Our results demonstrated that FPR, BHB, SCC, DSCC, lactose, and milk yield collectively offer a biologically meaningful, non-invasive framework for the dairy cows’ welfare assessment. These findings support the already present initiative of development of decision support tools that integrate biomarker data with housing, management, and behavioural monitoring to create early warning systems for welfare decline [10,43]. The strong associations observed between milk biomarkers and health, nutritional, and metabolic parameters are consistent with Glatz-Hoppe et al. [61], who reported that routine milk testing can predict the metabolic status and disease risk at the herd level. These findings also align with Linstädt et al. [62], whose systematic review confirmed the validity and practical applicability of ABMs and advocated their integration into standardised welfare protocols.

### 4.3. Multivariate Insights: PCA and Clustering

The PCA revealed three biologically meaningful axes: a health–hygiene axis (PC1), a socio-behavioural axis (PC2), and a metabolic stress axis (PC3). These results align with multivariate frameworks showing that welfare emerges from complex interactions between metabolic, clinical, and behavioural domains [40]. PC1 underscored the role of SCC and DSCC as key drivers of health-related welfare deficits, consistent with recent findings linking these markers to both mastitis and reduced milk quality [27,28]. PC2 highlighted the influence of stockmanship and behavioural expression, with positive affective states associated with balanced nutrition and good human–animal relationships [35,63]. PC3 captured the welfare costs of metabolic imbalance, confirming associations between BHB, hygiene deficits, and altered emotional reactivity [16,54].

Cluster analyses supported these dimensions, identifying three farm typologies: feeding–metabolic, behavioural–comfort, and clinical–hygiene risk. Such profiles mirror those reported in large-scale welfare monitoring projects [52,64] and in recent studies comparing bulk milk quality across welfare levels [10]. Hierarchical clustering further confirmed that milk biomarkers, behavioural indicators, and clinical traits co-vary, supporting the use of integrated monitoring frameworks [10,38].

Together, these multivariate results demonstrated that welfare cannot be explained by single parameters but emerges from interconnected biological and behavioural domains. Integrating milk biomarkers with animal-based indicators, therefore, offers a powerful contribution to modern precision livestock farming, enabling early detection of risks and benchmarking of herd welfare.

## 5. Conclusions

This study shows that routine milk biomarkers can serve as robust, non-invasive indicators of dairy cow welfare. Variability in metabolic traits, udder health, and hygiene was consistently linked to animal-based outcomes such as feeding status, comfort, disease, human–animal interactions, and emotional expression. Multivariate analyses further identified three coherent welfare domains (metabolic–nutritional balance, behavioural–comfort, and clinical–hygiene risk) that distinguished farm profiles. By integrating milk analysis into welfare monitoring, farmers and advisors can detect problems earlier, make evidence-based decisions, and strengthen both the sustainability and social acceptability of dairy production.

## 6. Limitations

This study has several limitations. The sample included 37 farms from a single geographical region of Romania, which may limit generalisation to other breeds, systems, or climates. The cross-sectional design identified associations but did not establish causal relationships between milk biomarkers and welfare outcomes. Longitudinal studies are needed to explore temporal dynamics. Although the Welfare Quality^®^ protocol is comprehensive, it may not fully capture all behavioural or environmental dimensions. In addition, the relatively low variance explained by PCA suggests that unmeasured factors such as genetics, microclimates, or management practice also contributed. Although individual factors such as breed, lactation stage, parity, and diet were not directly controlled or explicitly modelled, their effects were inherently accounted for through herd-level averaging, as the unit of analysis was the farm.

## 7. Implications for Practice and Future Research

Despite its limitations, this study highlighted the value of milk biomarkers as integrative, non-invasive indicators of dairy cow welfare. Collecting milk individually from cows proved especially valuable, as it revealed within-herd variability that is often masked in bulk tank analyses. Incorporating routine milk analysis into monitoring frameworks could help farmers and veterinarians detect welfare risks earlier, reduce reliance on invasive or costly assessments, and support evidence-based management decisions. The combination of metabolic indicators (FPR, BHB, urea), udder health traits (SCC, DSCC, lactose), and hygiene measures (TPC) offers a multidimensional perspective that complements direct animal-based observations. From a practical standpoint, the adoption of in-line sensors and mid-infrared spectroscopy would allow continuous monitoring at both herd and individual levels, enabling faster responses to emerging welfare challenges. For future research, larger and more diverse datasets are needed to validate these associations across production systems, climates, and breeds. Longitudinal studies could clarify causal pathways linking metabolic imbalance, immune function, and behaviour. Moreover, integrating milk biomarkers with digital tools, such as behavioural sensors and machine learning algorithms, may facilitate predictive modelling of welfare risk. Embedding such approaches into certification schemes and policy frameworks could strengthen sustainable and socially acceptable milk production, ensuring benefits for farmers, animals, and consumers alike.

## Figures and Tables

**Table 1:** Milk biomarkers, udder health indicators, and production traits (mean ± SD; median and min-max).

Milk Biomarker	Mean ± SD	Median (Min–Max)
Fat (g/100 g)	3.95 ± 0.42	3.92 (3.06–4.81)
Protein (g/100 g)	3.57 ± 0.32	3.54 (2.90–4.26)
FPR	1.10 ± 0.10	1.11 (0.92–1.34)
Casein (g/100 g)	2.84 ± 0.25	2.83 (2.30–3.44)
Lactose (g/100 g)	4.76 ± 0.14	4.76 (4.44–5.01)
Urea (mg/dL)	22.75 ± 10.02	22.80 (5.77–44.79)
Acetone (mmol/L)	0.01 ± 0.02	0.00 (0.00–0.09)
BHB (mmol/L)	0.03 ± 0.04	0.02 (0.00–0.19)
DSCC (%)	60.81 ± 14.88	62.30 (30.00–86.30)
SCC (×1000/mL)	480.55 ± 237.10	452.60 (82.41–953.40)
TPC (×1000/mL)	247.24 ± 146.04	95.00 (10.00–2321.00)
Milk yield (L)	11.41 ± 3.08	11.00 (7.00–25.00)

FPR—fat-to-protein ratio, BHB—β-hydroxybutyrate, DSCC—differential somatic cell count, SCC—somatic cell count, TPC—total plate count, SD—standard deviation, Min—minimum, Max—maximum.

**Table 2:** Descriptive statistics for welfare principle and criteria scores in 37 dairy farms.

Principle and Criteria	Mean ± SD	Median (Min–Max)
Good feeding *	51.89 ± 27.93	43.61 (13.26–100.00)
Absence of prolonged hunger	49.81 ± 33.39	49.99 (7.05–100.00)
Absence of prolonged thirst	73.15 ± 31.84	100.00 (12.12–100.00)
Good housing *	54.87 ± 17.08	60.40 (18.02–82.75)
Comfort around resting	40.86 ± 16.52	45.12 (8.64–72.62)
Ease of movement	80.62 ± 30.24	100.00 (34.00–100.00)
Good health *	19.88 ± 10.59	17.16 (9.31–55.04)
Absence of diseases	10.46 ± 18.29	4.22 (0.00–100.00)
Absence of injuries	65.36 ± 16.79	67.17 (33.46–99.20)
Absence of pain induced by management procedures *	43.92 ± 24.22	28.00 (20.00–100.00)
Appropriate behaviour *	27.36 ± 11.13	24.46 (13.18–59.87)
Expression of other behaviours	11.89 ± 15.09	0.00 (0.00–67.15)
Expression of social behaviours	96.30 ± 2.87	96.19 (86.83–100.00)
Good human–animal relationship	60.04 ± 21.27	59.59 (25.87–100.00)
Positive emotional state	30.48 ± 15.40	28.60 (6.61–67.94)

SD—standard deviation, Min—minimum, Max—maximum. * Welfare principles

**Table 3:** Descriptive statistics for the animal-based measures in 37 dairy farms.

Measure	Mean ± SD	Median (Min–Max)
Duration of lying down movements (seconds)	4.99 ± 1.23	5.25 (2.50–7.20)
Lying down movements with collisions (%)	19.89 ± 17.11	17.00 (0.00–76.60)
Lying cows which lie partly outside the lying area (%)	1.55 ± 2.97	0.00 (0.00–13.30)
Cows with dirty flanks and upper legs (%)	55.39 ± 31.75	50.00 (5.00–100.00)
Cows with dirty lower legs (%)	95.70 ± 10.64	100.00(56.00–100.00)
Cows with dirty udders (%)	77.13 ± 25.75	86.25 (12.50–100.00)
Very lean cows (%)	19.41 ± 19.43	8.88 (0.00–64.40)
Non-lame cows (%)	84.87 ± 12.72	88.10 (50.00–100.00)
Severely lame cows (%)	3.32 ± 4.05	2.30 (0.00–15.30)
Moderately lame cows (%)	11.81 ± 10.64	7.70 (0.00–40.00)
Cows with at least one hairless patch and no lesion/swelling (%)	27.78 ± 20.36	21.30 (3.30–75.90)
Cows with at least one lesion/swelling (%)	9.50 ± 6.16	8.30 (0.00–24.10)
Cows with diarrhoea (%)	22.87 ± 21.71	16.40 (0.00–79.40)
Cows with increased respiratory rate (%)	6.64 ± 5.43	5.50 (0.00–29.70)
Cows with nasal discharge (%)	4.07 ± 4.32	3.20 (0.00–17.20)
Cows with no integument alteration, no hairless patch, and no lesion (%)	62.72 ± 24.12	67.70 (0.00–96.70)
Cows with ocular discharge (%)	4.12 ± 5.74	1.90 (0.00–27.80)
Cows with vulvar discharge (%)	2.78 ± 4.64	1.30 (0.00–23.80)
Mastitis (%)	32.34 ± 22.49	32.10 (0.44–100.00)
Downer cows (%)	4.41 ± 3.79	4.40 (0.00–20.00)
Mortality (%)	6.07 ± 7.38	2.50 (0.00–28.60)
Cows that can be approached between 50 cm and 1 m (%)	21.00 ± 19.28	15.00 (0.00–69.70)
Cows that can be approached by 50 cm but not touched (%)	41.26 ± 24.87	34.50 (0.00–90.80)
Cows that can be touched (%)	33.86 ± 32.59	18.70 (0.00–100.00)
Cows that cannot be approached (%)	3.87 ± 5.85	0.00 (0.00–19.30)
Frequency of displacements/cow per hour	0.05 ± 0.05	0.05 (0.00–0.20)
Frequency of butts/cow per hour	0.04 ± 0.03	0.04 (0.00–0.10)

SD—standard deviation, Min—minimum, Max—maximum.

**Table 4:** Descriptive statistics for each descriptor of the qualitative behaviour assessment in 37 dairy farms.

Descriptor	Mean ± SD	Median (Min—Max)
Tendency to be active	85.95 ± 17.23	93.00 (45.00–110.00)
Tendency to be agitated	36.97 ± 13.11	35.00 (20.00–75.00)
Tendency to be apathetic	30.59 ± 8.32	30.00 (20.00–55.00)
Tendency to be bored	60.97 ± 17.50	60.00 (30.00–100.00)
Tendency to be calm	77.43 ± 16.00	78.00 (40.00–110.00)
Tendency to be content	75.19 ± 18.44	75.00 (30.00–110.00)
Tendency to be distressed	38.65 ± 15.18	35.00 (20.00–80.00)
Tendency to be fearful	43.35 ± 17.29	40.00 (20.00–80.00)
Tendency to be friendly	71.89 ± 15.68	75.00 (45.00–100.00)
Tendency to be frustrated	42.51 ± 17.80	40.00 (20.00–79.00)
Tendency to be happy	75.08 ± 17.78	75.00 (40.00–100.00)
Tendency to be indifferent	55.68 ± 13.69	55.00 (25.00–80.00)
Tendency to be inquisitive	43.97 ± 20.41	37.00 (10.00–100.00)
Tendency to be irritable	37.51 ± 14.95	35.00 (20.00–80.00)
Tendency to be lively	75.03 ± 17.98	75.00 (30.00–100.00)
Tendency to be playful	67.11 ± 18.16	70.00 (20.00–96.00)
Tendency to be positively occupied	67.59 ± 23.82	70.00 (20.00–105.00)
Tendency to be relaxed	79.41 ± 15.96	75.00 (50.00–110.00)
Tendency to be sociable	74.95 ± 16.97	75.00 (40.00–105.00)
Tendency to be uneasy	31.68 ± 10.03	30.00 (20.00–65.00)

SD—standard deviation, Min—minimum, Max—maximum.

**Table 5:** Significant Spearman correlations between milk biomarkers and animal-based welfare parameters after FDR adjustment (FDR ≤ 0.05).

Biomarker/Production Trait	Associated Welfare Parameter	r_s_	*p*-Value adj.
Fat-to-protein ratio (FPR)	Good feeding	0.68	<0.001
	Absence of prolonged hunger	0.78	<0.001
	Absence of diseases	0.66	<0.001
	Good human–animal relationship	0.66	<0.001
	Very lean cows	−0.78	<0.001
	Diarrhoea	−0.79	<0.001
β-hydroxybutyrate (BHB) (mmol/L)	Comfort around resting	−0.53	0.01
	Absence of injuries	−0.51	0.014
	Lesions/swelling	0.44	0.046
	Dystocia	−0.64	<0.001
Milk yield (L)	Good housing	0.55	0.006
	Good health	0.48	0.022
	Ease of movement	0.44	0.045
	Content behaviour	0.44	0.042
	Mortality	−0.47	0.026
	Udder dirtiness	−0.54	0.007
SCC (×1000/mL)	Good health	−0.53	0.01
	Absence of diseases	−0.57	0.004
	Positive emotional state	−0.44	0.043
	Mastitis prevalence	0.51	0.013
	Udder dirtiness	0.56	0.005
	Lameness	0.55	0.006
DSCC (%)	Good housing	−0.6	<0.002
	Good health	−0.58	0.003
	Absence of injuries	−0.6	0.002
	Calmness (QBA)	−0.55	0.01
	Relaxation (QBA)	−0.62	0.008
	Happiness (QBA)	−0.57	0.004
	Sociability (QBA)	−0.56	0.009
Lactose (g/100 g)	Good health	0.49	0.02
	Inquisitive behaviour	0.49	0.02
	Calm (QBA)	0.48	0.026
	Happy (QBA)	0.47	0.027
	Content (QBA)	0.44	0.045
	Relaxed (QBA)	0.43	0.047
Fat (g/100 g)	Absence of disease	0.47	0.026
	Good human–animal relationship	0.5	0.017
	Diarrhoea	−0.61	0.001
	Distress behaviour	−0.49	0.019

r_s_—Spearman’s rank correlation coefficient, FDR—false discovery rate, *p*-value adj. ≤ 0.05 is significant.

## Data Availability

The original contributions presented in this study are included in the article/Supplementary Material. Further inquiries can be directed to the corresponding authors.

## Supplementary Materials

The following supporting information can be downloaded at: https://www.mdpi.com/article/10.3390/ani15213202/s1, Figure S1: Scree plot from Principal Component Analysis (PCA). Eigenvalues are plotted against the number of components, showing the proportion of variance explained; Figure S2A: Evaluation of the optimal number of clusters for K-means analysis: Calinski–Harabasz index; Figure S2B: Evaluation of the optimal number of clusters for K-means analysis: average silhouette width; Figure S2C: Evaluation of the optimal number of clusters for K-means analysis: gap statistic.
