# Supplementary material for: Integrative Multivariate Analysis of Milk Biomarkers, Productive Performance, and Animal Welfare Indicators in Dairy Cows
Source: Animals (Basel). 2025 Nov 3;15(21):3202. doi: 10.3390/ani15213202 (PMC12607959; doi:10.3390/ani15213202)
Supplement: Supplementary file 1 [file animals-15-03202-s001.zip › animals-3931790-supplementary.pdf]

animals

MDPI

Article

Integrative Multivariate Analysis of Milk Biomarkers, Productive Performance, and Animal Welfare Indicators in Dairy Cows

Daniela Elena Babiciu [1*], Florin Ioan Beteg[1], Mihai Cenariu[1], Anamaria Blaga Petean[1*], Sorin Marian Mârza[1], Eva Andrea Lazar[2], and Silvana Popescu [1]

Supplementary Material

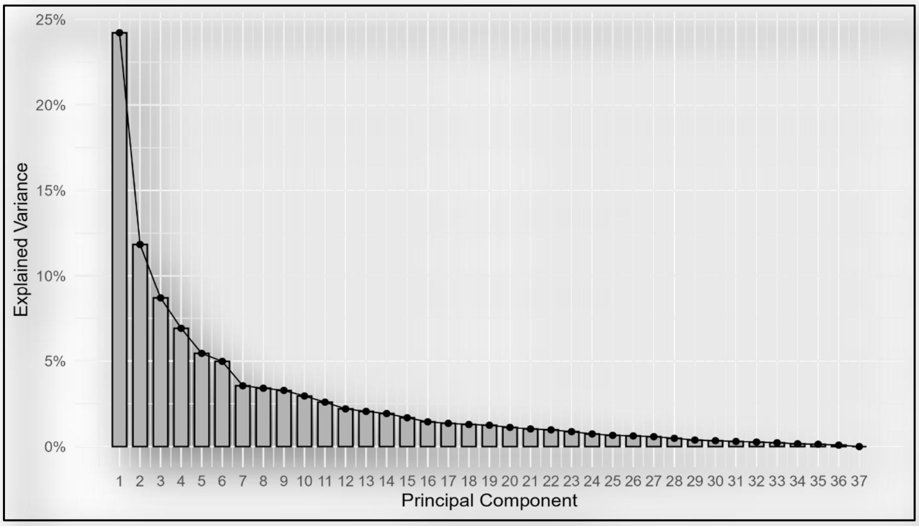

Figure S1. Scree plot from Principal Component Analysis (PCA). Eigenvalues are plotted against the number of components, showing the proportion of variance explained.

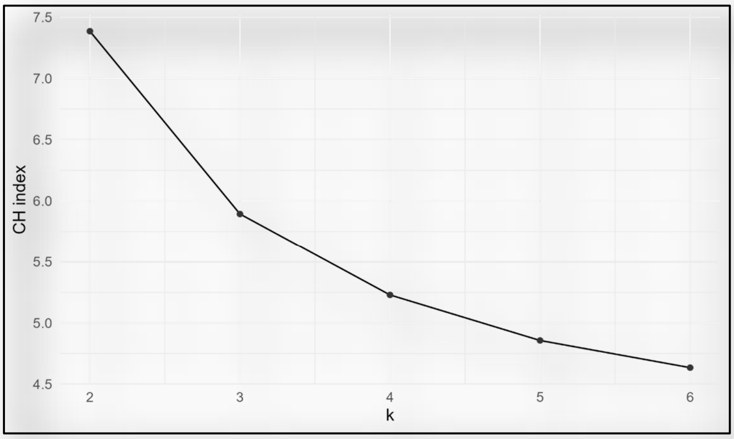

Figure S2A. Evaluation of the optimal number of clusters for k-means analysis: Calinski–Harabasz index.

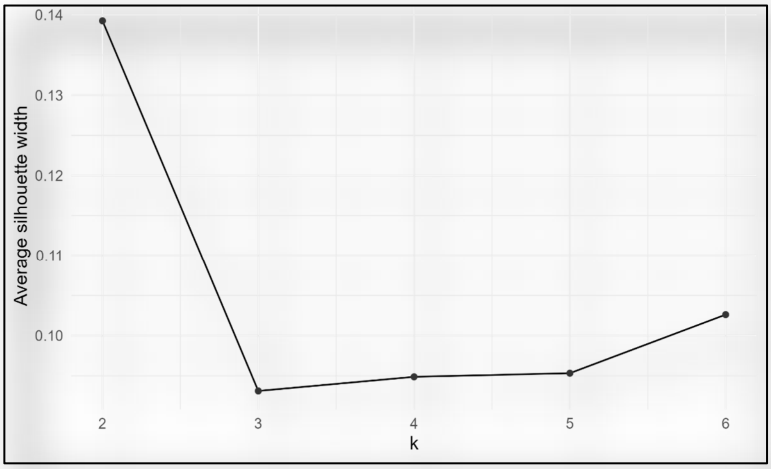

Figure S2B. Evaluation of the optimal number of clusters for k-means analysis: average silhouette width.

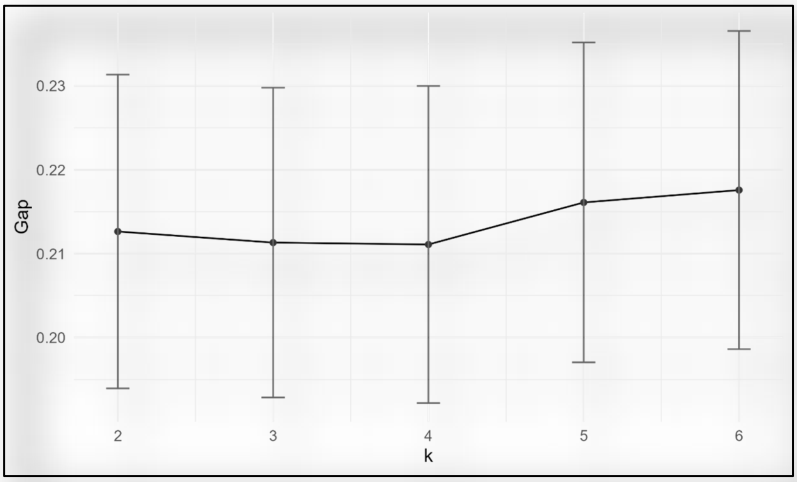

Figure S2C. Evaluation of the optimal number of clusters for k-means analysis: Gap statistic.
